# Stroke-Like Episodes and Cerebellar Syndrome in Phosphomannomutase Deficiency (PMM2-CDG): Evidence for Hypoglycosylation-Driven Channelopathy

**DOI:** 10.3390/ijms19020619

**Published:** 2018-02-22

**Authors:** Mercè Izquierdo-Serra, Antonio F. Martínez-Monseny, Laura López, Julia Carrillo-García, Albert Edo, Juan Darío Ortigoza-Escobar, Óscar García, Ramón Cancho-Candela, M Llanos Carrasco-Marina, Luis G. Gutiérrez-Solana, Daniel Cuadras, Jordi Muchart, Raquel Montero, Rafael Artuch, Celia Pérez-Cerdá, Belén Pérez, Belén Pérez-Dueñas, Alfons Macaya, José M. Fernández-Fernández, Mercedes Serrano

**Affiliations:** 1 Laboratori de Fisiologia Molecular, Departament de Ciències Experimentals i de la Salut, Universitat Pompeu Fabra, 08003 Barcelona, Spain; merce.izquierdo@upf.edu (M.I.-S.); julia.carrillo@upf.edu (J.C.-G.); albert.edo01@estudiant.upf.edu (A.E.); 2 Genetic Medicine and Rare Diseases Pediatric Institute, Hospital Sant Joan de Déu, 08002 Barcelona, Spain; afmartinez@hsjdbcn.org; 3 Unit of Child Neurology, Department of Pediatrics, Hospital Infantil Universitario Niño Jesús de Madrid, 28009 Madrid, Spain; laural.marin@hotmail.com (L.L.); lgutierrez.hnjs@salud.madrid.org (L.G.G.-S.); 4 Neuropediatric, Radiology and Clinical Biochemistry Departments, Hospital Sant Joan de Déu, 08002 Barcelona, Spain; jortigoza@sjdhospitalbarcelona.org (J.D.O.-E.); jmuchart@hsjdbcn.org (J.M.); rmontero@hsjdbcn.org (R.M.); rartuch@hsjdbcn.org (R.A.); bperez@hsjdbcn.org (B.P.-D.); 5 U-703 Centre for Biomedical Research on Rare Diseases (CIBER-ER), Instituto de Salud Carlos III, 08002 Barcelona, Spain; 6 Pediatric Department, Hospital Virgen de la Salud, 45004 Toledo, Spain; oscargarcam@hotmail.com; 7 Pediatric Neurology Unit, Pediatrics Department, Hospital Universitario Rio Hortega, 47012 Valladolid, Spain; rcanchoc@saludcastillayleon.es; 8 Neuropediatric Department, Pediatric Service, Hospital Universitario Severo Ochoa, Leganés, 28009 Madrid, Spain; maria.llanos@salud.madrid.org; 9 Statistics Department, Fundació Sant Joan de Déu, 08002 Barcelona, Spain; dcuadras@fsjd.org; 10 Centro de Diagnóstico de Enfermedades Moleculares (CEDEM), Universidad Autónoma de Madrid (UAM), U-746 Centre for Biomedical Research on Rare Diseases (CIBER-ER) Madrid, Instituto de Salud Carlos III, IdiPAZ, 28009 Madrid, Spain; cpcerda@cbm.csic.es (C.P.-C.); bperez@cbm.csic.es (B.P.); 11 Grup de Recerca en Neurologia Pediàtrica, Institut de Recerca Vall d’Hebron, Universitat Autònoma de Barcelona, Secció de Neurologia Pediàtrica, Hospital Universitari Vall d’Hebron, 08002 Barcelona, Spain; amacaya@vhebron.net

**Keywords:** ataxia, cerebellum, congenital disorders of glycosylation, magentic resonance Imaging (MRI), stroke-like, Ca_V_2.1 voltage-gated calcium channel

## Abstract

Stroke-like episodes (SLE) occur in phosphomannomutase deficiency (PMM2-CDG), and may complicate the course of channelopathies related to Familial Hemiplegic Migraine (FHM) caused by mutations in *CACNA1A* (encoding Ca_V_2.1 channel). The underlying pathomechanisms are unknown. We analyze clinical variables to detect risk factors for SLE in a series of 43 PMM2-CDG patients. We explore the hypothesis of abnormal Ca_V_2.1 function due to aberrant *N*-glycosylation as a potential novel pathomechanism of SLE and ataxia in PMM2-CDG by using whole-cell patch-clamp, *N*-glycosylation blockade and mutagenesis. Nine SLE were identified. Neuroimages showed no signs of stroke. Comparison of characteristics between SLE positive versus negative patients’ group showed no differences. Acute and chronic phenotypes of patients with PMM2-CDG or *CACNA1A* channelopathies show similarities. Hypoglycosylation of both Ca_V_2.1 subunits (α_1A_ and α_2α_) induced gain-of-function effects on channel gating that mirrored those reported for pathogenic *CACNA1A* mutations linked to FHM and ataxia. Unoccupied *N*-glycosylation site N283 at α_1A_ contributes to a gain-of-function by lessening Ca_V_2.1 inactivation. Hypoglycosylation of the α_2_δ subunit also participates in the gain-of-function effect by promoting voltage-dependent opening of the Ca_V_2.1 channel. Ca_V_2.1 hypoglycosylation may cause ataxia and SLEs in PMM2-CDG patients. Aberrant Ca_V_2.1 *N*-glycosylation as a novel pathomechanism in PMM2-CDG opens new therapeutic possibilities.

## 1. Introduction

Phosphomannomutase deficiency (PMM2-CDG), caused by mutations in *PMM2* (*601785OMIM), is the most frequent congenital disorder of *N*-linked glycosylation [1,2]. Patients with PMM2-CDG develop cerebellar atrophy and a cerebellar syndrome (axial and limb ataxia, abnormal eye movements, dysarthria, cognitive impairment, and long-term disability) [3,4].

Stroke-like episodes (SLE) are among the acute neurological complications that may occur in PMM2-CDG patients. SLE are typically triggered by infections and have been described in about 20–55% of patients with PMM2-CDG [5,6,7,8]. The term SLE was initially coined for MELAS (Mitochondrial myopathy, Encephalopathy, Lactic acidosis, and Strokelike episodes) syndrome to stress the non-ischemic origin of their events [9], but it may be used for different neurological diseases that are associated with focal deficits that mimic clinically, but not neuroradiologically, an ischemic injury.

SLE in PMM2-CDG patients are characterized by confusional status, mono- or hemiparesis, and sometimes, epileptic seizures. The underlying pathomechanisms are not fully understood, and clinical guidelines helping their diagnosis, and prevention and treatment are missing, resulting in a very stressful situation for families and doctors.

Although coagulation abnormalities are very frequent in PMM2-CDG, no significant correlations have been found between the presence of thrombophilia and the occurrence of SLE [5,6,7,8]. Besides, the majority of patients with PMM2-CDG and SLE did not show vascular occlusions on magnetic resonance angiography, the affected brain lesions did not reveal restricted diffusion, or did not follow well-defined vascular territories on MRI (magnetic resonance imaging) [5,6,7,8,9,10,11].

SLE may also complicate the course of other neurological diseases such as channelopathies related to Familial Hemiplegic Migraine (FHM), a paroxysmal neurological disease caused by mutations in *CACNA1A* (encoding the neuronal pore-forming Ca_V_2.1 channel α_1A_ subunit), *ATP1A2* (encoding the Na^+^, K^+^-ATPase pump α_2_ subunit) or *SCN1A* (encoding the neuronal Na_V_1.1 channel α_1_ subunit). As for PMM2-CDG, the pathogenesis of SLE in channelopathies is still not fully understood [12,13]. Interestingly, some clinical, neuroimaging and neurophysiological features of PMM2-CDG patients and *CACNA1A* mutated patients are similar [13], including not only SLE, but also ataxia, ocular motor disturbances, and cerebellar atrophy on MRI [14].

Ion channels are highly *N*-glycosylated and we are not aware of studies on the effect of an abnormal glycosylation of the α_1A_ channel subunit on Ca_V_2.1 functional expression and gating. As other high-voltage activated (HVA) Ca^2+^ channels, Ca_V_2.1 also contains at least the regulatory α_2_δ and β subunits [15]. Impaired glycosylation of the auxiliary α_2_δ subunit reduces the functional expression of different HVA channels (including Ca_V_2.1) in the plasma membrane [16,17,18], but its relevance (if any) on Ca_V_2.1 channel gating is unknown. Moreover, increased or decreased Ca_V_2.1-mediated Ca^2+^ influx has been related to different clinical features. Thus, FHM relates to gain-of-function mutations on *CACNA1A*, while ataxia is associated with the alteration of a narrow window of Ca^2+^ homeostasis in Purkinje cells due to either *CACNA1A* loss- or gain-of-function mutations [19].

We report on 9 SLE in a series of 43 PMM2-CDG patients and analyze different clinical, laboratory, and neuroimaging variables to detect potential SLE risk factors. We demonstrate the clinical similarities between the acute and chronic phenotypes of patients with PMM2-CDG or patients carrying *CACNA1A* mutations, establishing a novel link between both diseases. With those evident clinical similarities and knowing that gain-of-function *CACNA1A* mutations can lead to both paroxysmal neurological symptoms and cerebellar symptoms including cerebellar atrophy (such as progressive or congenital ataxia) [19], we explore the hypothesis of increased Ca_V_2.1 activity due to deficient *N*-glycosylation as an underlying biological reason, offering a potential novel pathomechanism of SLE and cerebellar syndrome in PMM2-CDG.

## 2. Results

Forty-three children and young adults with PMM2-CDG were included. Seven patients had nine SLE (mean age 7.5 ± 4.8 years, range 3.3–15.0 years) representing 17.9% of the patients (SLE positive group). Using our SLE definition, two clinical episodes were excluded: one caused by a true cerebrovascular event as confirmed by the MRI, and one caused by an epileptic seizure occurring at the onset of an infection process of the brain.

Thirty-two PMM2-CDG patients (mean age 16.2 ± 9.6, range 4.2–38 years) did not have SLE and were used as PMM2-CDG control group (SLE negative group).

### 2.1. Stroke-Like Episodes

Table 1 summarizes the age at the SLE, molecular studies, clinical presentation and follow-up, neurophysiological and neuroimaging findings of all patients.

In six SLE, a mild-to-moderate head trauma was identified as a potential trigger. SLE-related neurological symptoms started after a symptom-free interval ranging between 1 and 24 h. For three patients suffering from infections it was not possible to exactly quantify the symptom-free interval. Three out of nine episodes were characterized by focal neurological deficits and three patients presented with clinical seizures. All patients had abnormal consciousness (Supplementary Video 1).

Almost all patients developed hyperthermia at presentation leading to a laboratory work-up for a possible underlying infection or inflammatory process (Table 1). Acute phase proteins and complete blood counts were normal for all patients with SLE after head trauma.

**Figure 1:**
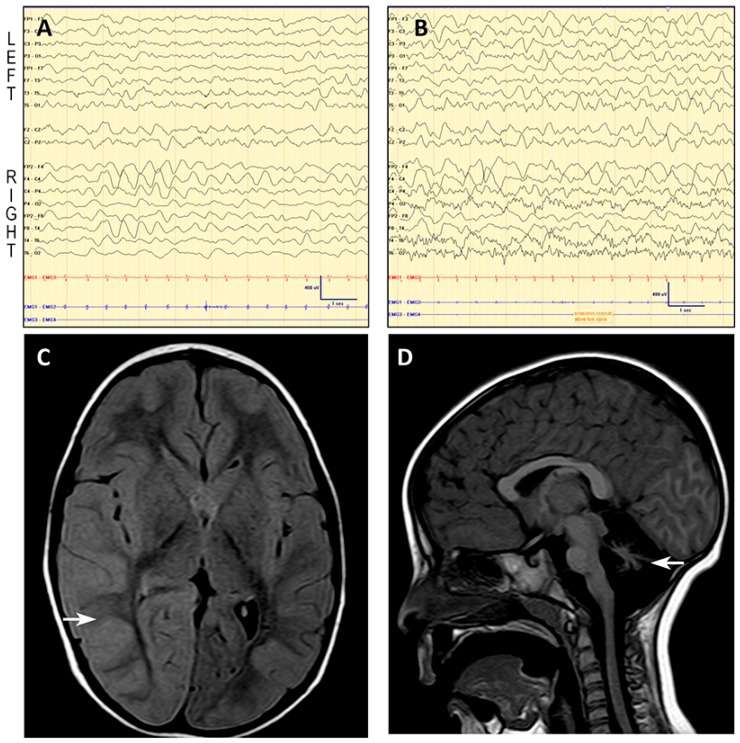
Patient 1: EEG and MRI. EEG shows asymmetric (right) slow background trace with moderately low voltage in temporal regions (**A**,**B**), and frontal intermittent rhythmic delta activity (FIRDA) in the right hemisphere, coherent with the edema found on the MRI and the left hemiparesis. Below, axial fluid attenuation inversion recovery (FLAIR) MR image reveals cortical diffuse edema in the right hemisphere, mainly in the parieto-occipital region (**C**, see arrow). Midsagittal T1-weighted MR image of the same patient shows a small cerebellar vermis with enlarged interfolial spaces representing atrophy and secondary enlargement of the fourth ventricle (**D**, see arrow).

In seven SLE a brain MRI was performed during the first 72 h. Neuroimaging showed the preexisting cerebellar atrophy with absence of new findings in all but Patient 1 (Table 1), who showed diffuse cortical swelling and FLAIR-hyperintense signal in the right hemisphere with no restricted diffusion, suggestive of vasogenic edema (Figure 1).

EEG revealed normal findings in six out of nine studies (Table 1). On the second day after SLE onset, the EEG of Patient 1 showed an asymmetric (right) slow background activity with moderately low voltage in temporal regions, and frontal intermittent rhythmic delta activity (FIRDA) in the right hemisphere (Figure 1).

Antiepileptic drugs were used in six of nine SLE with two aims: to treat irritability using midazolam (two out of four cases), and to control or prevent epileptic seizures using levetiracetam, midazolam, valproic acid, phenytoin, and diazepam (Table 1). Neither anticoagulants nor antiaggregants were used in eight of nine episodes, as there was no evidence of a procoagulant situation. Patient 4 developed a deep venous thrombosis during her second SLE. Her coagulation tests revealed abnormal findings that required fibrinogen substitution.

Most of patients recovered completely within one week. Three patients, however, experienced a more delayed recovery (Table 1). After SLE onset, Patient 1 and Patient 6 developed episodic tonic upgaze that progressively decreased in frequency and disappeared three and five weeks after onset, respectively (Supplementary Video 2).

### 2.2. SLE Versus SLE Negative Group

Statistical analysis of clinical, laboratory, and neuroimaging variables showed no significant differences between PMM2-CDG patients with and without SLE. Epidemiological and clinical data are shown in Table 2.

### 2.3. Literature Review Comparing Clinical Presentations between Patients with PMM2-CDG and Patients with CACNA1A Mutations

Table 3 compares the acute and chronic clinical presentations that have been reported in patients with pathogenic variants in *CACNA1A* and *PMM2*, respectively. The similarities in acute and chronic neurological presentations suggest a possible common underlying pathomechanism for SLE.

### 2.4. Deficient N-Glycosylation Alters Ca_V_2.1 Functional Expression, Activation, and Inactivation

**Figure 2:**
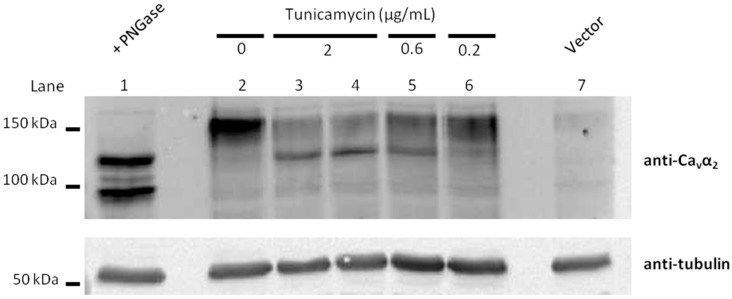
Hypoglycosylation levels of the Ca_V_ regulatory α_2_δ subunits under different concentrations of tumicamycin. Immunoblot analysis of glycosylated fragments of α_2_δ subunits heterologously expressed in HEK293 cells (along with the α_1A_ Ca_V_2.1 pore-forming channel subunit and β_3_ regulatory subunit), treated or not with increasing concentrations of tunicamycin (0.2, 0.6 and 2 μg/mL, as indicated). The extent of α_2_δ glycosylation was identified by using antibody anti-α_2_ (1:500 dilution, Sigma D219, St. Louis, MO, USA) (top panels) and is shown as the difference in molecular weight between the glycosylated and unglycosylated forms. Molecular weight markers are indicated on the left. PNGase F was also added to protein extraction from tunicamycin-untreated cells in order to identify unglycosylated forms in vitro. Upper bands potentially corresponding to glycosylated α_2_δ progressively decrease as the concentration of tunicamycin increases, which in turn promotes the appearance of a lower band potentially corresponding to unglycosylated α_2_δ (lanes 2 to 6). In vitro PNGase F treatment (lane 1) shows two main bands potentially corresponding to unglycosylated α_2_δ (upper band) and unglycosylated α_2_ (lower band), as previously reported [26].Protein extraction from HEK293 cells transfected only with the vector plasmid was included as negative control (lane 7). For each experimental condition, the protein sample was probed with anti-tubulin (1:2000 dilution, Sigma T6074) (bottom panel) as loading control.

Whole-cell patch-clamp recordings obtained from HEK293 cells expressing Ca_V_2.1 channels composed of α_1A_, β_3_ and α_2_δ_1_ subunits, showed that tunicamycin treatment (which specifically blocks the first step of *N*-glycosylation by inhibiting *N*-acetylglucosamine transferase in the endoplasmic reticulum, Figure 2), reduced Ca_V_2.1 Ca^2+^ currents in a concentration-dependent manner.

**Figure 3:**
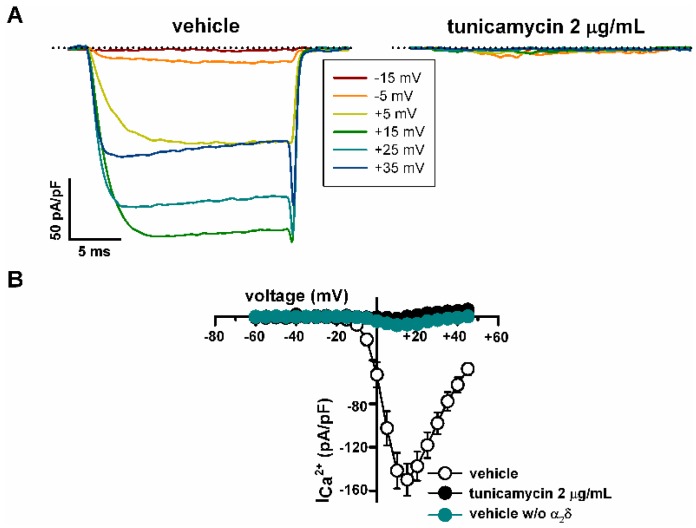
Inhibition of *N*-glycosylation strongly reduces functional expression of Ca_V_2.1 channels containing β_3_ and α_2_δ_1_ subunits, heterologously expressed in HEK293 cells. (**A**) Representative current traces elicited by 20 ms depolarizing pulses from −80 mV to the indicated voltages (inset), illustrating the significant (see Results) reduction in Ca^2+^ current amplitude through Ca_V_2.1 channels induced by tunicamycin (2 μg/mL) versus vehicle (DMSO) treatment. The zero current level is indicated by dotted lines. (**B**) Average Ca^2+^ current density-voltage relationships for DMSO-treated cells in the presence (open circles, *n* = 27) and in the absence (*w*/*o*) (filled cyan circles, *n* = 7) of the α_2_δ_1_ subunit, and for tunicamycin-treated cells (filled black circles, *n* = 6). No significant differences on the maximal current density between cells treated with tunicamycin and cells lacking α_2_δ_1_ were observed (Kruskal-Wallis test followed by Dunn post hoc test).

Thus, 2 μg/mL tunicamycin almost abolished Ca_V_2.1 Ca^2+^ currents (Figure 3A right panel, and B) compared to cells treated with the vehicle (DMSO) (Figure 3A left panel, and B). Such tunicamycin effect was mimicked by removal of the regulatory α_2_δ_1_ subunit (Figure 3B). Under these experimental conditions, maximal Ca^2+^ current density was reduced from −149.8 ± 14.9 pA/pF (*n* = 27) to −1.9 ± 1.8 pA/pF by tunicamycin (*n* = 6) (*p* < 0.001) and to −7.8 ± 2.9 pA/pF in the absence of the α_2_δ_1_ subunit (*n* = 7) (*p* < 0.001) (Kruskal-Wallis test followed by Dunn post hoc test) (Figure 3B).

**Figure 4:**
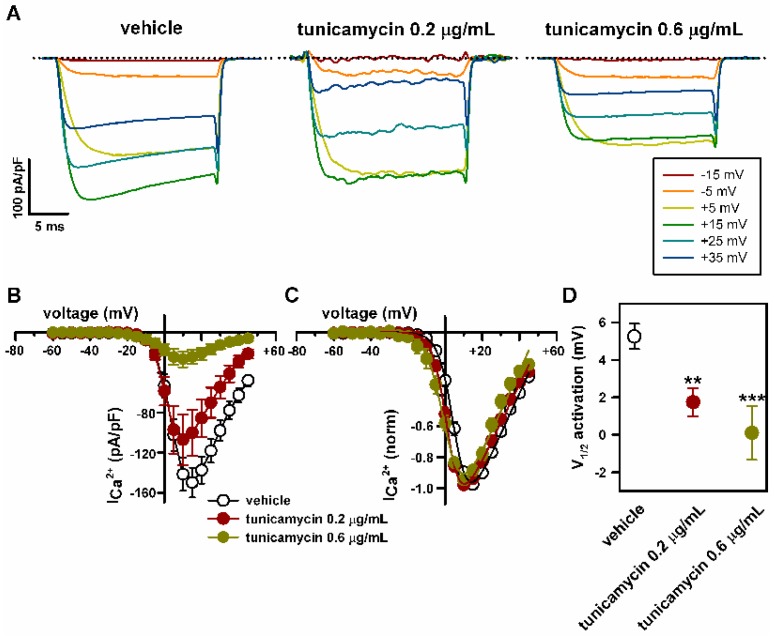
Reduction in the levels of *N*-glycosylation favors voltage-dependent activation of Ca_V_2.1 channels heterologously expressed in HEK293 cells. (**A**) Current traces elicited by 20 ms depolarizing pulses from −80 mV to the indicated voltages (inset), illustrating the shift of Ca_V_2.1 activation to lower depolarization, induced by 0.2 and 0.6 μg/mL tunicamycin. Dotted lines indicate the zero current level. Average Ca^2+^ current density-voltage relationships (**B**) and I-V curves normalized to peak Ca^2+^ current density (**C**) for Ca_V_2.1 channels expressed in DMSO-treated cells (open circles, *n* = 27) and in cells treated with 0.2 μg/mL (filled red circles, *n* = 13) or 0.6 μg/mL tunicamycin (filled green circles, *n* = 16). (**D**) Reduction in V_1/2_ for Ca_V_2.1 channel activation (estimated from normalized I-V curves, as indicated in Materials and Methods) induced by 0.2 (*n* = 13) and 0.6 μg/mL (*n* = 16) tunicamycin. ** *p* < 0.01 and *** *p* < 0.001 versus the control condition (vehicle, *n* = 27; Kruskal-Wallis test followed by Dunn *post hoc* test).

At lower concentrations, a smaller decrease of α_2_δ_1_ glycosylation by tunicamycin was found (Figure 2) and the effect on the amplitude of Ca_V_2.1 Ca^2+^ currents was less important, with reductions in peak current density to −107.03 ± 25.1 pA/pF (*n* = 13) (*p* > 0.05) and to −26.7 ± 7.9 pA/pF (*n* = 16) (*p* < 0.001) induced by 0.2 and 0.6 μg/mL tunicamycin, respectively (Kruskal-Wallis test followed by Dunn post hoc test) (Figure 4A,B). The Ca_V_2.1 voltage-dependent activation curve was left-shifted and the potential for half-maximal channel activation (V_1/2_ activation, directly related to the energy necessary to open the channel) was significantly decreased in response to both 0.2 and 0.6 μg/mL tunicamycin treatments (by ~3.5 mV and ~5 mV, respectively) (Figure 4C,D). Consistently, the maximum current amplitude was elicited by depolarizing pulses to +15 mV or +10 mV for control (vehicle) or tunicamycin treatments, respectively (Figure 4B,C).

**Figure 5:**
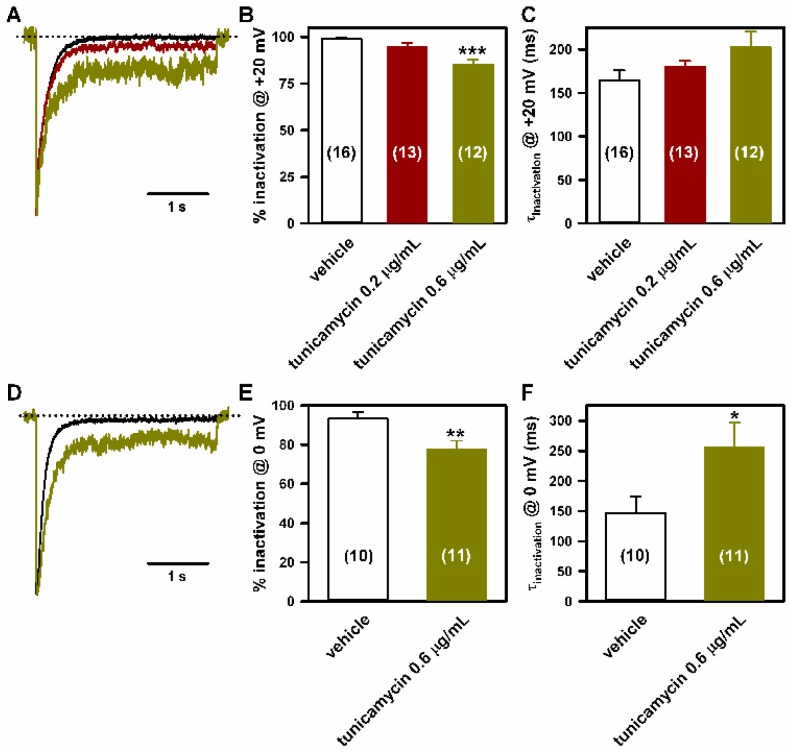
Inactivation of Ca_V_2.1 channels heterologously expressed in HEK293 cells is impaired by lowering *N*-glycosylation. Representative current traces (normalized to the corresponding peak amplitude) illustrating the effects of 0.2 (red trace) and 0.6 μg/mL (green traces) tunicamycin on Ca_V_2.1 inactivation when compared to DMSO-treated cells (black traces), in response to a 3 s depolarizing pulse to +20 mV (**A**) or 0 mV (**D**). The zero current level is indicated by dotted lines. (**B**,**E**) Average Ca^2+^ current inactivation (in %) at the end of these 3 s depolarizing pulses obtained from HEK293 cells expressing Ca_V_2.1 channels and treated with DMSO (vehicle) or with tunicamycin (0.2 and 0.6 μg/mL). Data are expressed as the mean ± SEM of the number of experiments shown in brackets. *** *p* < 0.001 (Kruskal-Wallis test followed by Dunn post hoc test) and ** *p* < 0.01 (Student’s *t*-test) versus the control (vehicle) condition. (**C**,**F**) Average τ inactivation values of Ca^2+^ currents through Ca_V_2.1 channels expressed in HEK293 cells treated with DMSO (vehicle) or with tunicamycin (0.2 and 0.6 μg/mL), elicited by a 3 s depolarizing pulse to +20 mV or 0 mV, as indicated. Data are expressed as the mean ± SEM of the number of experiments shown in brackets. * *p* < 0.05 versus the control (vehicle) condition (Mann-Whitney *U*-test).

Tunicamycin (0.6 μg/mL) also impaired the inactivation of Ca_V_2.1 Ca^2+^ currents: compared to the control (vehicle) condition, the degree of inactivation at the end of 3s depolarizing pulses to +20 (Figure 5A,B) or 0 mV (Figure 5D,E) was lower (by ~14% and ~16%, respectively); and there was a tendency for slow Ca_V_2.1 inactivation, which reached statistical significance for Ca^2+^ currents elicited by depolarization to 0 mV (Figure 5A,C,D,F).

### 2.5. Mutation of α_1A_ Potential Glycosylation Site Mimics Tunicamycin Effect on Ca_V_2.1 Inactivation

**Figure 6:**
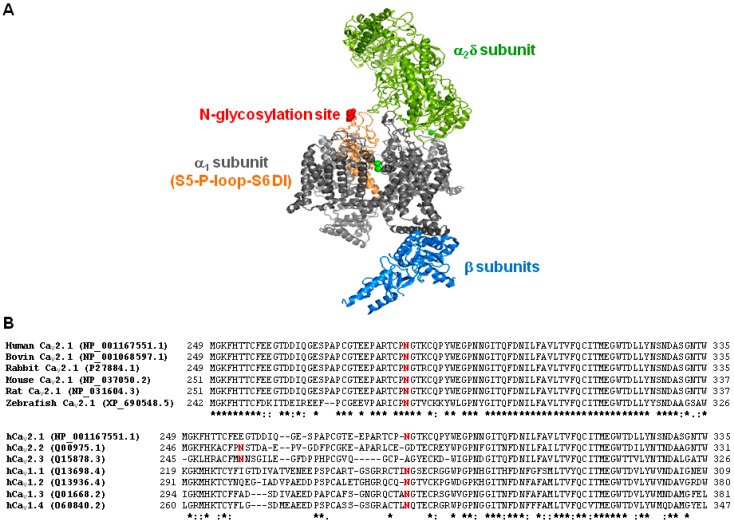
Location and conservation of the N283 amino acid residue. (**A**) Illustration showing the location of the potential glycosylation site at residue N283 (in red) at the extracellular P-loop between S5 and S6 transmembrane segments in domain I of the pore forming α_1A_ subunit (in orange). The structure of the Ca_V_1.1 complex, containing α_1_ (in grey), β (in blue) and α_2_δ (in green) subunits, was used as model (PDB 5GJV). (**B**) Sequence alignment of P-loop regions at domain I (DI) of Ca_V_2.1 channel α_1A_ subunits from different organisms (as indicated) (top), and sequence alignment of P-loop-DI α_1_ regions of human high-voltage activated Ca^2+^ channels belonging to the Ca_V_2.x and Ca_V_1.x families (bottom). Alignments were performed with Clustal Omega (www.ebi.ac.uk/Tools/msa/clustalo/, access on 30 November 2017). Potential sites for *N*-glycosylation (including N283 at the human Ca_V_2.1 channel) are shown in red. Asterisk means identical residues; colon and period indicates the existence of conservative and semi-conservative amino acid substitutions, respectively.

To evaluate whether abnormal glycosylation of the α_1A_ channel subunit may contribute to the effect produced by tunicamycin on Ca_V_2.1 biophysical properties, we first searched for potential *N*-glycosylation sites in the sequence of the human α_1A_ subunit (GenBank No. O00555) by using Uniprot Knowledgebase (www.uniprot.org, access on 30 November 2017). One single glycosylation site at residue N283, located in the extracellular P-loop region at domain I (DI) of α_1A_, was identified (Figure 6A). This asparagine residue is highly conserved through evolution (Figure 6B, top). In addition, potential glycosylation of a single N residue at P-loop-DI is a trait shared by all human high-voltage activated (HVA) Ca^2+^ channels (Figure 6B, bottom).

**Figure 7:**
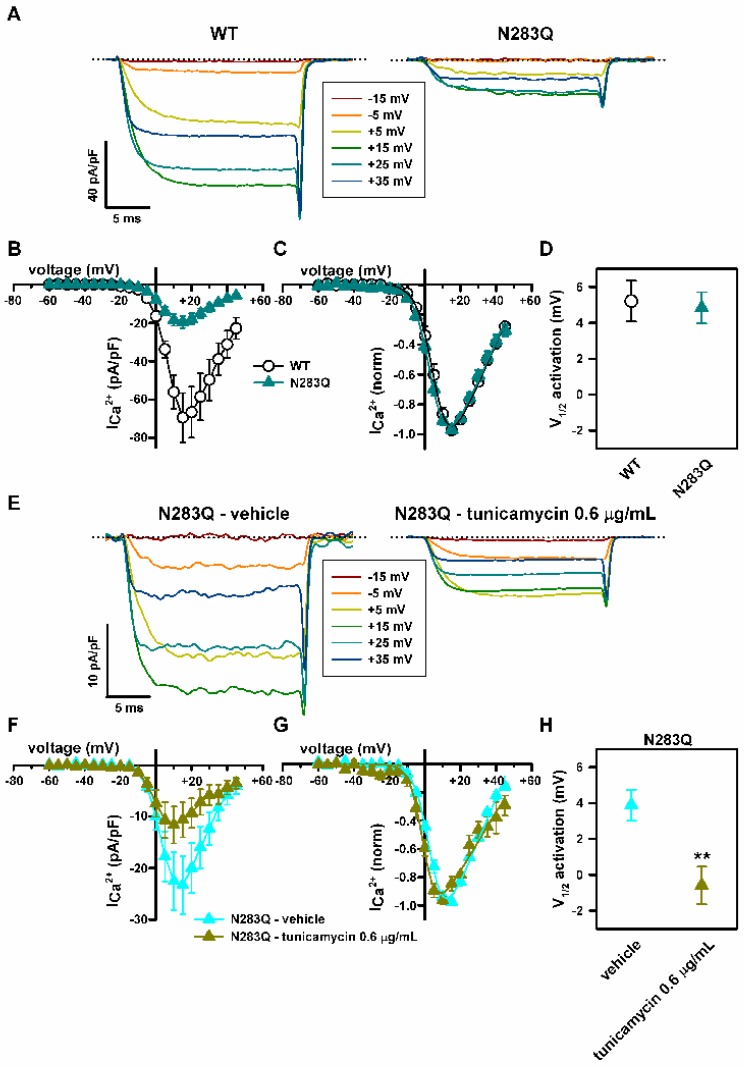
N283Q glycosylation site mutation reduces Ca^2+^ current density through Ca_V_2.1 channels heterologously expressed in HEK293 cells, without altering their voltage-dependent activation. (**A**) Current traces elicited by 20 ms depolarizing pulses from −80 mV to the indicated voltages (inset) illustrating the decrease in Ca^2+^ current density through Ca_V_2.1 channels containing the mutation at the α_1A_ glycosylation site N283, which exhibit voltage dependence of activation similar to that of WT channels. Dotted lines mark the zero current level. Average Ca^2+^ current density-voltage relationships (**B**) and normalized I-V curves (**C**) for WT (open circles, *n* = 13) and N283Q (filled dark cyan triangles, *n* = 19) Ca_V_2.1 channels. N283Q reduces maximal Ca^2+^ current density (obtained by membrane depolarization to +15 mV) from −69.4 ± 12.9 pA/pF (*n* = 13) to −19.5 ± 3.2 pA/pF (*n* = 19) (*p* < 0.001, Mann-Whitney *U*-test). (**D**) N283Q mutation has no significant effect on the V_1/2_ for Ca_V_2.1 channel activation (estimated from normalized I-V curves shown in **C** as indicated in Materials and Methods, *p* = 0.798, Student’s *t*-test). (**E**) Current traces elicited by 20 ms depolarizing pulses from −80 mV to the indicated voltages (inset) illustrating the shift of activation to lower depolarization induced by 0.6 μg/mL tunicamycin on N283Q mutant Ca_V_2.1 channels containing β_3_ and α_2_δ_1_ subunits. The zero current level is indicated by dotted lines. Average Ca^2+^ current density-voltage relationships (**F**) and normalized I-V curves (**G**) for N283Q mutant Ca_V_2.1 channels expressed in DMSO-treated cells (filled cyan triangles, *n* = 10) and in cells treated with 0.6 μg/mL tunicamycin (filled green circles, *n* = 8). Peak Ca^2+^ current density through N283Q Ca_V_2.1 channels after vehicle (DMSO) and tunicamycin treatments were −23.3 ± 5.6 pA/pF (*n* = 10) and −11.6 ± 3.6 pA/pF (*n* = 8), respectively (*p* = 0.06, Student’s *t*-test). (**H**) Reduction in V_1/2_ for activation of Ca_V_2.1 channels composed by N283Q mutant α_1A_, β_3_ and α_2_δ_1_ subunits (estimated from normalized I-V curves shown in (**G**) as indicated in Materials and Methods) produced by 0.6 μg/mL tunicamycin (*n* = 8). ** *p* < 0.01 versus the control condition (vehicle, *n* = 10; Student’s *t*-test).

Hence, we compared the biophysical properties of Ca_V_2.1 channels composed by wild-type (WT) or N283Q glycosylation mutant α_1A_, β_3_ and α_2_δ_1_ subunits, heterologously expressed in HEK293 cells. Peak Ca^2+^ current density for N283Q mutant Ca_V_2.1 channels were ~72% lower than for WT channels (Figure 7A,B). N283Q did not alter Ca_V_2.1 voltage-dependent activation (Figure 7C,D). Interestingly, tunicamycin (0.6 μg/mL) shifted V_1/2_ activation for N283Q Ca_V_2.1 channels to less depolarized potentials (by ~4.5 mV) (Figure 7E–H), in line with its gain-of-function effect on WT Ca_V_2.1 activation (Figure 4A–D).

**Figure 8:**
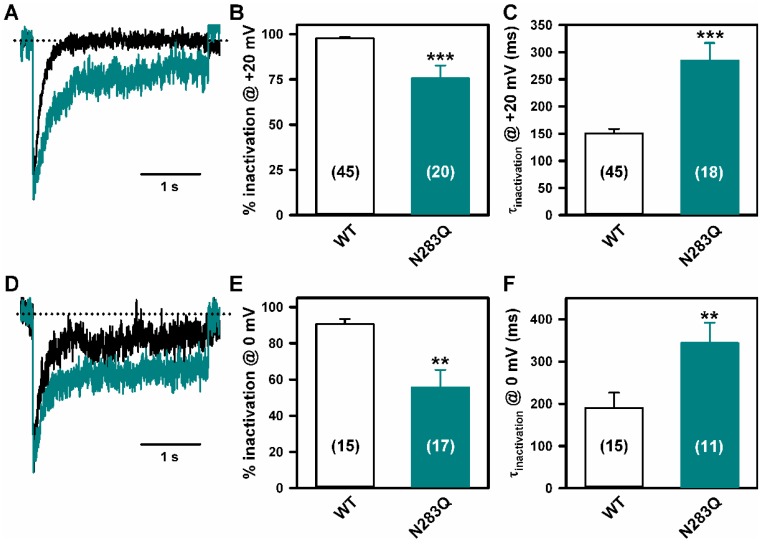
N283Q glycosylation site mutation lessens Ca_V_2.1 channel inactivation. Ca^2+^ current traces (normalized to the corresponding peak amplitude) illustrating differential inactivation of WT (black traces) and N283Q (cyan traces) Ca_V_2.1 channels, in response to a 3 s depolarizing pulse to +20 mV (**A**) or 0 mV (**D**). Dotted lines indicate the zero current level. (**B**,**E**) Average Ca^2+^ current inactivation (in %) at the end of these 3s depolarizing pulses obtained from HEK293 cells expressing either WT or N283Q Ca_V_2.1 channels. Data are expressed as the mean ± SEM of the number of experiments shown in brackets (*** *p* < 0.001 and ** *p* < 0.01 versus WT, Mann-Whitney *U*-test). (**C**,**F**) Average τ inactivation values of Ca^2+^ currents through WT (open bars) and N283Q (cyan bars) Ca_V_2.1 channels expressed in HEK293 cells, elicited by a 3 s depolarizing pulse to +20 mV or 0 mV, as indicated. Data are expressed as the mean ± SEM of the number of experiments shown in brackets (*** *p* < 0.001 and ** *p* < 0.01 versus WT, Mann-Whitney *U*-test).

Finally, Ca^2+^ currents through N283Q mutant Ca_V_2.1 channels in response to 3 s depolarizing pulses to either +20 mV (Figure 8A–C) or 0 mV (Figure 8D–F), showed lower (~22% and ~35%, respectively) and slower inactivation than currents through WT channels.

## 3. Materials and Methods

### 3.1. Patients

Patients with a molecular diagnosis of PMM2-CDG were recruited from the hospitals collaborating in the Spanish PMM2-CDG Network, and were followed from May 2014 to December 2016.

We used the following SLE definition: “Acute event consisting of sudden onset of a focal neurological deficit, irritability or decreased consciousness that may associate with seizures, headache or other transient symptoms, in the absence of another diagnosis explaining these symptoms”. The definition does not include neurophysiological and neuroimaging findings. Patients with “true” ischemic stroke on MRI were excluded.

We collected epidemiological and molecular data, potential triggers of SLE, clinical findings that occurred shortly before the episode, symptoms during the episode and the recovery process, and laboratory, neurophysiological (EEG), and neuroimaging (computed tomography (CT) and MRI) findings. Laboratory studies included blood cell counts, liver enzymes, acute phase reactants and coagulation parameters. Clinical severity of cerebellar syndrome was assessed through International Cooperative Ataxia Rating Scale (ICARS) [25].

To compare PMM2-CDG patients with SLE (SLE group) and without SLE (SLE negative group), we used epidemiological and molecular data, laboratory findings, personal history of epilepsy, personal or familial history of vascular events, and neuroimaging findings, using the midsagittal vermis relative diameter (MVRD) [25,27].

Biochemical studies were performed during the SLE. For the patients without SLE, the most abnormal liver function and coagulation laboratory values were included in the statistical analysis. For this comparison, four patients with early death in young childhood (before 2 years) were excluded, because of their severe systemic involvement.

Molecular studies had been carried out in all PMM2-CDG patients enrolled. Genetic analysis was performed in the “Centro de Diagnóstico de Enfermedades Moleculares” in Madrid. Total mRNA and genomic DNA were isolated from venous whole blood or patient-derived fibroblasts using a MagnaPure system following the manufacturer’s protocol (Roche Applied Science, Indianapolis). Mutational analysis was performed by genomic DNA analysis both in patients’ and parents’ samples to assure that both changes are on different alleles and to rule out the presence of a large genomic rearrangement. In some cases, the effect on splicing was analyzed by cDNA profile analysis. The primers used for cDNA and genomic DNA amplifications were designed using the ENSEMBL database (http://www.ensembl.org/index.html, ENSG00000140650) and GenBank accession number NM_000303.2.

### 3.2. Literature Review

To compare the cerebellar syndrome and SLE in PMM2-CDG patients and the phenotype related to *CACNA1A* mutated patients, we searched PubMed for articles on *PMM2*, PMM2-CDG, CDG-Ia, congenital disorders of glycosylation, *CACNA1A*, familial hemiplegic migraine, episodic ataxia type 2 and spinocerebellar ataxia type 6 that have been published between 1 January 1980, and 31 May 2017, and used different combinations of these terms.

### 3.3. cDNA Constructs

cDNA of the human voltage-gated Ca^2+^ (Ca_V_2.1) channel α_1A_ wild-type (WT) subunit (originally cloned into a pCMV vector) was a gift from Professor J. Striessnig (University of Innsbruck, Austria). cDNAs of the rabbit α_2_δ_1_ and rat β_3_ regulatory subunits (subcloned into a pcDNA3 expression vector) were gifts from Dr. L. Birnbaumer (National Institutes of Health, Durham, NC, USA). Ca_V_2.1 N283Q mutant channel was generated by site-directed mutagenesis of the human α_1A_ cDNA (GenScript Corporation, Piscatway, NJ, USA). All cDNA clones used in this study were sequenced in full to confirm their integrity.

### 3.4. Heterologous Expression

HEK293 cells were transfected using a linear polyethylenimine (PEI) derivative, the polycation ExGen500 (Fermentas Inc., Hanover, MD, USA) as previously reported (eight equivalents PEI/3.3 μg DNA/dish) [28]. Transfection was performed using the ratio for α_1A_ (WT or N283Q), β_3_, α_2_δ_1_, and EGFP (transfection marker) cDNA constructs of 1:1:1:0.3. When required, α_2_δ_1_ cDNA was replaced for pcDNA3 empty vector.

### 3.5. Inhibition of N-Glycosylation in Live Cells and Western Blot

One day after transfection, cells were grown for another day in the presence of tunicamycin (0.2, 0.6 or 2 μg/mL) or vehicle (dimethyl sulfoxide, DMSO). At the end of the treatment, cells were washed with 1× PBS, returned to the incubator, and electrophysiological recordings were performed 4 h later at room temperature (22–24 °C). For Western Blot analysis of glycosylated fragments of heterologously expressed α_2_δ_1_ subunit, after 4 h from recovery of the above mentioned tunicamycin or vehicle (DMSO) treatments, HEK293 cells were lysed on ice in lysis buffer (50 mM Tris-HCl, pH 7.5, 150 mM NaCl, 0.5% *v*/*v* Nonidet P-40, 5 mM EDTA, 1 mM DTT, 10 mM β-glycerolphosphate, 0.1 mM Na_3_VO_4_, 1 μg/mL pepstatin, 2 μg/mL aprotinin, 0.1 mM phenylmethylsulfonyl fluoride) containing protease inhibitors (Complete Mini protease inhibitor cocktail, Roche). Lysates were vortexed for 15 min at 4 °C and centrifuged at 4 °C for 7 min at 13,500× *g* to pellet debris. Next, protein concentrations of cleared lysates were determined using Pierce BCA protein assay kit (Thermo Scientific, Madrid, Spain). Cell lysates (20 µg of proteins) were first incubated under denaturing conditions (0.5% SDS and 40 mM DTT) at 100 °C for 10 min, and then incubated in presence or absence of 500 units of peptide-*N*-glycosidase F (PNGase F, New England Biolab, Ipswich, MA, USA) during 1 h at 37 °C, according to the manufacturer’s instructions. Incubated lysates were denatured to inactivate PNGase by incubation for 10 min at 80 °C, with 4× LDS Sample Buffer (Life Technologies, Carlsbad, CA, USA) and 10× sample reducing agent (Life Technologies). Samples were electrophoresed on an 8% SDS-polyacrylamide denaturating gel, transferred to a nitrocellulose membrane with iBlot (Invitrogen, Madrid, Spain), and probed with anti-rabbit Ca_V_α_2_ antibody (1:500 dilution, Sigma D219) and anti-tubulin (1:2000 dilution, Sigma T6074) as a loading control, and mouse secondary antibody (GE Healthcare, Piscataway, NJ, USA). Signal was detected with the SuperSignal West Pico Chemiluminescent Substrate (Thermo Scientific). Blots were visualized with the ChemiDoc XRS documentation system (Bio-Rad, Hercules, CA, USA).

### 3.6. Electrophysiology

Ca^2+^ currents (I_Ca_^2+^) through Ca_V_2.1 channels were measured using the whole-cell configuration of the patch-clamp technique as described in detail previously [28]. In brief, pipettes had a resistance of 2–3 MΩ when filled with a solution containing (in mM): 140 CsCl, 1 EGTA, 4 Na_2_ATP, 0.1 Na_3_GTP, and 10 HEPES (pH 7.2–7.3 and 290–300 mOsmol/L). The external bath solution contained (in mM): 140 tetraethylammonium-Cl (TEACl), 3 CsCl, 2.5 CaCl_2_, 1.2 MgCl_2_, 10 HEPES and 10 d-glucose (pH 7.4 and 300–310 mOsmol/L). Recordings were obtained with a D-6100 Darmstadt (List Medical, Darmstadt-Eberstadt, Germany) or an Axopatch 200A amplifier (Molecular Devices, San Jose, CA, USA), and the pClamp8 or pClamp10.5 software (Molecular Devices) was used for pulse generation, data acquisition and subsequent analysis.

Maximal inward Ca^2+^ current (I_Ca_^2+^) densities in response to 20 ms depolarizing pulses were measured from cells clamped at −80 mV, as detailed in previous report [28]. To evaluate the effect of tunicamycin and mutation N283Q on Ca_V_2.1 voltage-dependent activation, normalized current-voltage (I–V) relationships were individually fitted with the modified Boltzmann equation, as previously reported [28]:

(1) $$I\;=\;\frac{Gmax\left(V-Vrev\right)}{1+e^{\frac{-\left(V-V1/2\;act\right)}{kact}}}$$

where *I* is the peak current, *G_max_* is the maximal conductance of the cell, *V* is the membrane potential, *V_rev_* is the extrapolated reversal potential of *I*_Ca_^2+^, *V*_1__/__2_ *_act_* is the voltage for half-maximal current activation, and *k_act_* is the slope factor of the Boltzmann term.

Time constant for Ca_V_2.1 inactivation (τ_inactivation_) under the different experimental conditions was calculated after single exponential fits of the *I*_Ca_^2+^ inactivation phase during a 3 s pulse from a holding potential of −80 mV to a test potential of +20 mV or 0 mV. The degree of *I*_Ca_^2+^ inactivation (in %) at the end of these 3 s depolarizing pulses was also measured.

### 3.7. Ethics Statement

This study was approved by the Research and Ethics Committee of the “Sant Joan de Déu Hospital (SJDH)” (Internal code PIC-108-14) (Internal code PIC-108-14). Parents gave their written informed consent. Samples and data were obtained in accordance with the Helsinki Declaration of 1964, as revised in October 2013 (Fortaleza, Brazil).

### 3.8. Statistical Analysis

Statistical analysis was performed using Program R 3.2 (Vienna, Austria). Numerical variables are compared between groups by means of Mann-Whitney’s *U*-test (non-parametric), and categorical variables by Fisher’s exact test. For electrophysiological analysis, data are presented as the means ± S.E.M. and statistical tests included Kruskal-Wallis test followed by Dunn post hoc test, Student’s *t*-test or Mann-Whitney *U*-test, as appropriate. Differences were considered significant if *p* < 0.05.

## 4. Discussion and Conclusions

In this multicenter cohort of 43 PMM2-CDG patients, we found a SLE incidence of 16.2% (7/39), slightly lower than previously reported (20–55%) [5,6,7]. Underdiagnosis due to the lack of defined clinical criteria for SLE, might explain such lower incidence. This situation also makes the differentiation from other more common complications of PMM2-CDG—such as epileptic seizures and true ischemic events—very challenging. To overcome this problem, we proposed and applied a SLE clinical definition.

Our results suggest two main trigger factors for SLE in PMM2-CDG: head trauma and viral infection. The association between cranial traumatisms and the occurrence of SLE in PMM2-CDG patients has been occasionally reported [5]. However, the role of head trauma and hyperthermia in triggering encephalopathic episodes is also described in other inborn errors of metabolism and child neurology conditions [29,30,31,32]. Here we report on six SLE occurring after mild accidental head trauma, a common situation in children with ataxia and hypotonia. Why only a minority of PMM2-CDG patients experiencing mild head trauma during their life develop SLE is currently unknown.

In our cohort, MRI during SLE revealed diffuse cortical edema in the parieto-occipital region of right hemisphere in Patient 1 (Figure 1), whereas no acute brain injuries were observed in the other eight SLE, as reported in many patients [8,20].

In PMM2-CDG different underlying pathomechanisms have been suggested for SLE, being hypoperfusion or ischemia the most discussed. However, they do not completely explain the nature and temporal course of the neuronal dysfunction [8,10]. In our PMM2-CDG cohort, no vascular injury was seen on brain MRI. Besides, no differences in coagulation factors were found between PMM2-CDG patients with and without SLE (Table 2).

Three patients showed abnormal EEG findings, including asymmetrical background activity with FIRDA, a characteristic finding of severe encephalopathy, but nonspecific for SLE (Figure 1), supporting the need of EEG monitoring in SLE and the use of antiepileptic drugs to treat neuronal dysfunction [8].

Although rarely reported [20], our results show that hyperthermia without any sign of infection in blood or CSF is consistently present during SLE, presumably secondary to hypothalamic thermoregulatory dysregulation. Although contribution of hyperthermia to cerebral/cerebellar damage has not been documented in PMM2-CDG, aiming at normothermia appears as a reasonable goal in SLE, in line with general neuroprotective recommendations in stroke.

Analysis of epidemiological, clinical, laboratory, and neuroimaging findings did not reveal differences between PMM2-CDG patients with and without SLE. Regarding the genotype, all kind of pathogenic variants were distributed among both groups.

Similarities in clinical presentation encompass both the acute and chronic manifestations of patients with PMM2-CDG and *CACNA1A* channelopathies (Table 3). Those in the acute phenotype led to the not previously explored hypothesis of a similar underlying pathomechanism for FHM episodes (related to *CACNA1A* gain-of-function mutations) and SLE in PMM2-CDG. The characteristics of episodic encephalopathic crises after minor head trauma associated with seizures, hemiparesis, hyperthermia, altered consciousness and reversible hemispheric swelling on brain MRI were common features of both disorders [12,13]. Also, a chronic course including cerebellar ataxia, nystagmus, and episodes of tonic upgaze has been described in patients with Ca_V_2.1 (*CACNA1A*) channelopathy [33], and in PMM2-CDG patients recovering from severe SLE as shown by this study (Video 2). Interestingly, in patients with *CACNA1A*-linked FHM, verapamil or acetazolamide may improve the symptoms and prevent recurrences [22,23,24,34]. This observation is of great importance because the similarity between the two diseases may be an argument to explore a potentially beneficial therapeutic use of these drugs in PMM2-CDG patients.

As a novel pathophysiological approach, we explored the hypothesis of abnormal Ca_V_2.1 function due to aberrant *N*-glycosylation as the potential cause of SLE in PMM2-CDG. We studied the effect of hypoglycosylation of heterologously expressed Ca_V_2.1 channels on their functional expression and gating. Our results suggest that α_2_δ_1_ hypoglycosylation induced by strong inhibition of *N*-glycosylation with 2 μg/mL tunicamycin may mediate the reduction in Ca^2+^ current density through Ca_V_2.1 channels. This agree with previous reports showing that: (1) glycosylation of specific asparagine residues at α_2_δ increases cationic current density through distinct Ca_V_ channels (including Ca_V_2.1) [16,17,18], (2) mutation of several α_2_δ consensus *N*-glycosylation sites disrupt its cell surface expression and diminish protein stability [18], which may reduce the number of functional Ca_V_ channels in the membrane by destabilizing the interaction between α_2_δ and pore-forming α_1_ subunits [16,17,18]. At lower tunicamycin concentrations (0.2 and 0.6 μg/mL) the reduction of Ca_V_2.1 Ca^2+^ current density is less important and a double gain-of-function effect is observed: 1) the Ca_V_2.1 voltage-dependent activation is favored, as indicated by the tunicamycin-induced shift of the current activation curve towards less depolarized potentials (by ~3.5–5 mV); and 2) the Ca_V_2.1 channel inactivation is impaired due to a lower degree of inactivation (that allows a persistent Ca^2+^ influx at the end of long (seconds) depolarizing pulses) and the slowing of inactivation kinetic.

A gain-of-function in the voltage-dependence of Ca_V_2.1 activation is the common feature described for all FHM-linked *CACNA1A* mutations that have been functionally analyzed, both in heterologous expression systems and in excitatory neurons from FHM Ca_V_2.1 knock-in mice: they reduce the voltage threshold of channel activation by 5.6–20.9 mV [28,35,36,37,38,39,40,41]. Besides, for some *CACNA1A* mutations, a relationship between impaired Ca_V_2.1 inactivation and a greater severity in the clinical phenotype (including progressive cerebellar or congenital ataxia) has been described [41,42,43,44].

Despite the association with current density reduction, increase in Ca_V_2.1 activity due to both favored voltage-dependent activation and impaired inactivation results in higher Ca^2+^ influx into the cell in response to stimuli of physiological relevance in neurons, such as either single or trains of action potential [41]. An augment in intracellular Ca^2+^ influx leads to the specific increase in cortical excitatory neurotransmission that promotes the generation and propagation of cortical spreading depression, which correlates with the neurological symptoms (auras) and triggers the headache phase that are typical for FHM [36,37,38,39,45,46,47,48]. In the cerebellum, enhanced Ca_V_2.1 activity favors the generation of somatic action potentials and dendritic Ca^2+^ spikes in Purkinje cells (PCs). This induces PCs hyperexcitability and, hence, mild constant cerebellar ataxia in a FHM knock-in mouse model [19,46].

Our functional analysis of the N283Q mutant Ca_V_2.1 channel α_1A_ subunit suggests that N283 glycosylation is essential for proper Ca_V_2.1 inactivation. To our knowledge, this is the first study on the importance of *N*-glycosylation at residues located at the P-loop-DI region of pore-forming α_1_ subunits in the biophysical properties of HVA Ca_V_ complexes formed by α_1_, β and α_2_δ. Only for low-voltage activated Ca_V_3.2 channels it has been shown that α_1_ subunit hypoglycosylation slowed inactivation kinetic [49,50].

Since α_1A_ mutation N283Q does not affect the voltage-dependence of Ca_V_2.1 opening (Figure 7A–D), the tunicamycin-induced gain-of-function effect on channel activation seems to be independent of aberrant α_1A_ subunit glycosylation, and is most probably due to α_2_δ hypoglycosylation. Supporting this observation, tunicamycin treatment of cells expressing the Ca_V_2.1 channel formed by the N283Q mutant α_1A_, β_3_ and α_2_δ_1_ subunits induces a reduction in the V_1/2_ for channel activation of similar magnitude to that induced on the wild-type (WT) channel (Figure 4 and Figure 7E–H). It is well known that α_2_δ subunits not only affect the surface expression of HVA Ca_V_ channels but also their function. α_2_δ effect on Ca_V_ activation most likely depends on the particular pore-forming α_1_ subunit of the channel. α_2_δ_1_ favored the opening of Ca_V_1.2 (α_1C_) channels by shifting their activation 10 mV to more hyperpolarized voltages [51,52]. Accordingly, the presence of α_2_δ_1_ glycosylation mutants impaired Ca_V_1.2 activation by voltage [18]. On the contrary, α_2_δ_1_ impaired the opening of Ca_V_2.3 (α_1E_) channels by depolarizing their voltage-dependent activation (by 7–12 mV) [53]. The latter observation matches our data showing that tunicamycin-induced hypoglycosylation favors the activation of another Ca_V_2 family member (Ca_V_2.1) containing the regulatory α_2_δ subunit along with either WT or N283Q glycosylation mutant α_1A_ subunit (Figure 4 and Figure 7E–H).

In summary, we propose a clinical definition for SLE to avoid misclassification of acute events in PMM2-CDG patients. The lack of well-defined diagnostic criteria and the high level of suspicion required for the diagnosis may result in SLE underdiagnosis. Our data suggest that mild cranial trauma and infections may trigger SLE in PMM2-CDG patients. Similarities in the clinical presentation of SLE in PMM2-CDG and *CACNA1A*-related encephalopathies suggest a possible common underlying pathomechanism. Our findings show that hypoglycosylation of different Ca_V_2.1 subunits promotes gain-of-function effects that are similar to those induced by *CACNA1A* pathogenic variants linked to FHM and different forms of ataxia. Hence, our results support the hypothesis that aberrant Ca_V_2.1 *N*-glycosylation may cause not only a cerebellar syndrome, but also SLE in PMM2-CDG patients. This is not to the detriment of the possible contribution of other hypoglycosylated proteins in the phenotypic expression of this disease, as we are aware that is difficult to extrapolate total cell hypoglycosylation to the effects on specific glycosylation sites at Ca_V_2.1 channel subunits. Clarifying SLE pathogenesis in PMM2-CDG may prove paramount to develop prophylactic or therapeutic strategies for this acute and stressful complication.

## Figures and Tables

**Table 1:** Molecular, clinical, electrophysiological and neuroimaging details from stroke-like episodes.

Patient/Episode	Sex/Age	Molecular Findings	ICARS	Trigger	Free Symptom Period	Initial Clinical Presentation	Body Temperature (°C)	C-reactive Protein/PCT	EEG	Neuroimaging (<72 h)	Treatment	Duration of Symptoms (h)	Recovery
1	M 3 yr 11 mo	p.V44A p.Q33Stop	NA	Head trauma	3 h	Lethargy, epileptic seizures, left hemiparesis	38.5	Normal	Asymmetric slow background, right hemisphere FIRDA epileptic activity	MRI: Asymmetric hemispheric vasogenic edema	LEV, PHE, MDZ	96	Tonic upgaze some days after discharge Left hemiparesis until 4 months later
2	M 15 yr	p. V44A p.R123G	NA	Head trauma	24 h	Somnolence, vomiting	37.4	No data available	Normal	MRI: No significant changes	No	48	Complete recovery
3	F 5 yr	p.T237M IVS7-9T > G	46	Head trauma	4 h	Somnolence, vomiting	37.5	Normal	Normal	No significant changes	No	7	Complete recovery
4-I	F 3 yr 6 mo	p.L32R IVS-1G > C	8	Head trauma	1 h	Irritability and lethargy	38.0	Normal	Normal	No significant changes	No	3	Complete recovery
4-II	F 14 yr 5 mo			Enterovirus	NA	Dysarthria, dysphasia, irritability, left hemiparesis	38.0	Normal	Normal	CT angiography: No significant changes	LEV	36	Complete recovery
5-I	F 7 yr 4 mo	p.D65Y IVS7-9T > G	56	Head trauma	12 h	Irritability, lethargy, aphasia and dysphagia	38.6	No data available	Normal	MRI: No significant changes	Risperidone	168	Complete recovery
5-II	F 10 yr 2 mo			Head trauma	2 h	Irritability and lethargy	38.5	Normal	Normal	MRI: No significant changes	MDZ	24	Complete recovery
6	F 3 yr 3 mo	p.P113L p.F207S	NA	Influenza virus infection	NA	Epileptic seizures, irritability and lethargy	38.9	38.0 mg/L 1.04 ng/mL	Asymmetric slow background, epileptic activity	CT: No significant changes	LEV, PHE, MDZ	264	Tonic upgaze deviation and irritability until 1 month later. Fully recovered 7 months later.
7	M 5 yr 8 mo	p.D65Y p.R141H	65	Upper respiratory viral infection	NA	Lethargy, epileptic seizures, left arm monoparesis	38.5	11.4 mg/L 24.7 ng/mL	Asymmetric slow background, no epileptic activity	MRI: No significant changes	VPA, LEV, DZP, MDZ	144	Distal weakness left arm, fully recovered in 2 weeks
Mean values/SD	7 yr 7 mo/ 4 yr 10 mo			6 out of 9 Head trauma	13.0/8.4		38.2/0.5		3 out of 9 Abnormal EEG	1/9 Hemispheric edema		87.8 h/86.9 h	
Range	3 yr 3 mo/15 yr				3–24 h		37.4–38.9					3–264 h	

M: male; F: female; yr: years; mo: months; ICARS: International Cooperative Ataxia Rating Scale; NA: Not available, difficult to determine; PCT: procalcitonin; FIRDA: Frontal intermittent rhythmic delta activity; LEV: levetiracetam; PHE; phenytoin; MDZ: midazolam; VPA: valproic acid; DZP: diazepam.

**Table 2:** Comparison of clinical and epidemiological data between PMM2-CDG patients with and without SLE.

		SLE Positive (SD)	SLE Negative (SD)	*p*-Value
Number of patients/episodes		7/9	32	
Age (years)		13.7 (11.6) *	16.2 (9.6)	0.46
Sex (Male:Female)		3:4	21:11	0.19
Liver function	AST (UI/mL) Normal values 2–34 UI/mL	378 (317.3)	172 (235.5)	0.61
	ALT (UI/mL) Normal values 2–36 UI/mL	277 (220.6)	216 (327.1)	0.70
Coagulation	PT (%) Normal values 80–120%	95.4 (13.1)	95.9(24.0)	0.82
	aPTT (seconds) Normal values 23–35 seconds	33.3 (4.6)	31.5 (6.0)	0.53
	F IX (%) Normal values 50–120%	49.5 (14.9)	75.2 (25.0)	0.17
	F XI (%) Normal values 50–120%	35.9 (15.1)	59.3 (35.5)	0.28
	AT III (%) Normal values 60–120%	43.0 (14.1)	51.2 (26.8)	0.82
	Protein C (%) Normal values 60–140%	38.6 (17.2)	83.4 (129.9)	0.46
	Protein S (%) Normal values 60–140%	52.9 (22.6)	63.5 (17.5)	0.31
Vascular events	Positive personal history	1/7	3/32	0.51
	Positive familial history	1/7	2/32	0.41
Vermis Midsagittal Relative Diameter (MRI)		0.48	0.41	0.44
Seizures	Febrile seizures	2/7	4/32	0.23
	Epilepsy	2/7	6/32	0.58

* Age at the time of the present study; SD: Standard deviation; AST: aspartate transaminase; ALT: Alanine transaminase; PT: prothrombin time; aPTT: activated partial thromboplastin time; F IX: coagulation factor IX; F XI: coagulation factor XI; ATIII: antithrombin III. Included values for the statistical studies are for the SLE group, values during the episode, and for those “SLE negative” patients, most abnormal values among all the different liver function tests (maximal AST and ALT) and coagulation analysis (lowest coagulation factor activities).

**Table 3:** Findings in patients carrying mutations in *CACNA1A* compared to findings in PMM2-CDG patients, including our cohort.

Gene	*CACNA1A*	*PMM2*
**Acute: stroke-like episodes**		
Prevalence	21% [8], >70 cases reported [14].	18 to 40–50%, >49 cases reported [20].
Age at onset	At any age between 1 to 73 years old [21].	Any age, frequently between 3 to 6 years old [5,8,11].
Trigger/Onset of encephalopathy after trigger	Minor head trauma [22], infections, diagnostic procedures (cerebral or coronary angiography), physical activity [21]; Immediately or 2–3 h after the trigger [14].	Infections, head trauma, angiography, alcohol ingestion [5]; Minutes to hours after the trigger.
Prodromi	Severe headache, yawning, truncal unsteadiness [22].	None
Focal deficits	Hemiparesis, dysphasia, nystagmus, dyskinetic limb movement [12,13,14].	Hemiparesis, dysphasia, dysphagia, conjugated eye deviation, blindness [5,8,11].
Epilepsy/EEG findings	Hemi-clonic or hemi-tonic convulsions/Globally slow background activity, spikes, spike-and-slow wave complexes, slow wave bursts, photoparoxysmal response [14].	Clonic convulsions/Low voltage pattern in affected areas, asymmetric slow background activity with moderately low frontal intermittent rhythmic delta activity (FIRDA) in the affected hemisphere [8].
Autonomic signs	High fever, recurrent vomiting [14].	High fever, recurrent vomiting [11].
MRI findings during the episode	Normal, ischemic lesion with prominent perifocal edema, or panhemispheric edema [14,23].	Normal or asymmetric hemispheric cytotoxic edema [8,11].
Duration/number of episodes	Few minutes to 10 days/1–11 episodes [14,21].	1h to several months/1–2 episodes [5,11].
Recovery	24 h to months. Complete recovery in the majority of the patients, but residual hemiplegia possible [14,22].	1h to several months. Complete recovery in the majority of the patients, but exceptionally residual motor symptoms may persist [5].
Treatment	Analgesia, antiepileptic drugs, sleep, acetazolamide, verapamil [13,22,23,24].	IV hydration, antiepileptic drugs [5].
**Chronic: time between stroke-like episodes**		
Ataxia	Episodic attacks of cerebellar dysfunction lasting from 5 minutes to 5 h [23].	Persistent ataxia [1,2,4,5,11].
Abnormal eye movement	Saccadic eye movements, downgaze nystagmus, strabismus, tonic upgaze episodes [22].	Strabismus, nystagmus, tonic upgaze episodes [4,20,25].
Other neurologic manifestations	Developmental disability, dysarthria, migraine with aura, motor stereotypies, benign paroxysmal torticollis [14,21].	Developmental disability, hypotonia, dysarthria [4,20,25].
MRI	Cerebellar vermis atrophy, mild delay in white matter myelination [22].	Atrophy of the cerebellar hemispheres and vermis [25], pontine hypoplasia [11].

## Supplementary Materials

Supplementary materials can be found at http://www.mdpi.com/1422-0067/19/2/619/s1.
