# Supplementary material for: Stroke-Like Episodes and Cerebellar Syndrome in Phosphomannomutase Deficiency (PMM2-CDG): Evidence for Hypoglycosylation-Driven Channelopathy
Source: Int J Mol Sci. 2018 Feb 22;19(2):619. doi: 10.3390/ijms19020619 (PMC5855841; doi:10.3390/ijms19020619)
Supplement: Supplementary file 1 [file ijms-19-00619-s001.zip › supplementary-proof/supplementary.docx]

**Stroke-Like Episodes and Cerebellar Syndrome in Phosphomannomutase Deficiency (PMM2-CDG): Evidence for Hypoglycosylation-Driven Channelopathy**

Mercè Izquierdo-Serra ^1,†^ Antonio F. Martínez-Monseny ^2,†^, Laura López ^3^, Julia Carrillo-García ^1^, Albert Edo ^1^ Juan Darío Ortigoza-Escobar ^4^, Óscar García ^5^, Ramón Cancho-Candela ^6^,
Llanos Carrasco-Marina ^7^, Luis G. Gutiérrez-Solana ^3^, Daniel Cuadras ^8^, Jordi Muchart ^4^,
Raquel Montero ^4^, Rafael Artuch ^4^, Celia Pérez-Cerdá ^9^, Belén Pérez ^9^, Belén Pérez-Dueñas ^4^, Alfons Macaya ^10^, José M. Fernández-Fernández ^1,^*, Mercedes Serrano ^2,4,^*

**Video 1.** Different phases of a stroke-like episode. Video 1 shows the clinical course of SLE for Patient 1 including initial irritability, lethargy on the second day, and, finally, a better state of alertness but left hemiparesis on the fourth day of evolution.

**Video 2.** Tonic upgaze. Patient 6 shows irritability and an episodic conjugated upwards movement of the eyes that she cannot control. The video was taken 16 days after the SLE.
